# The serine-threonine protein phosphatases that regulate the thiazide-sensitive NaCl cotransporter

**DOI:** 10.3389/fphys.2023.1100522

**Published:** 2023-02-15

**Authors:** Héctor Carbajal-Contreras, Gerardo Gamba, María Castañeda-Bueno

**Affiliations:** ^1^ Department of Nephrology and Mineral Metabolism, Instituto Nacional de Ciencias Médicas y Nutrición Salvador Zubirán, Mexico City, Mexico; ^2^ PECEM (MD/PhD), Facultad de Medicina, Universidad Nacional Autónoma de México, Mexico City, Mexico; ^3^ Molecular Physiology Unit, Instituto de Investigaciones Biomédicas, Universidad Nacional Autónoma de México, Mexico City, Mexico

**Keywords:** calcineurin (CaN), protein phosphatase (PP) 1, protein phophatase, with No lysine kinase (WNK), distal convoluted tubule, inhibitor 1 of protein phosphatase 1, tacrolimus, cyclosporin

## Abstract

The activity of the Na^+^-Cl^-^ cotransporter (NCC) in the distal convoluted tubule (DCT) is finely tuned by phosphorylation networks involving serine/threonine kinases and phosphatases. While much attention has been paid to the With-No-lysine (K) kinase (WNK)- STE20-related Proline Alanine rich Kinase (SPAK)/Oxidative Stress Responsive kinase 1 (OSR1) signaling pathway, there remain many unanswered questions regarding phosphatase-mediated modulation of NCC and its interactors. The phosphatases shown to regulate NCC’s activity, directly or indirectly, are protein phosphatase 1 (PP1), protein phosphatase 2A (PP2A), calcineurin (CN), and protein phosphatase 4 (PP4). PP1 has been suggested to directly dephosphorylate WNK4, SPAK, and NCC. This phosphatase increases its abundance and activity when extracellular K^+^ is increased, which leads to distinct inhibitory mechanisms towards NCC. Inhibitor-1 (I1), oppositely, inhibits PP1 when phosphorylated by protein kinase A (PKA). CN inhibitors, like tacrolimus and cyclosporin A, increase NCC phosphorylation, giving an explanation to the Familial Hyperkalemic Hypertension-like syndrome that affects some patients treated with these drugs. CN inhibitors can prevent high K^+^-induced dephosphorylation of NCC. CN can also dephosphorylate and activate Kelch-like protein 3 (KLHL3), thus decreasing WNK abundance. PP2A and PP4 have been shown in *in vitro* models to regulate NCC or its upstream activators. However, no studies in native kidneys or tubules have been performed to test their physiological role in NCC regulation. This review focuses on these dephosphorylation mediators and the transduction mechanisms possibly involved in physiological states that require of the modulation of the dephosphorylation rate of NCC.

## Introduction

The Na^+^-Cl^-^ cotransporter (NCC) is the most important apical route for Na^+^ reabsorption in the distal convoluted tubule (DCT) (Castañeda-Bueno and Ellison, 2022). The phosphorylation of its N-terminus by serine/threonine kinases positively modulates its activity in a critical manner, as shown by multiple *in vitro*, *ex vivo* and *in vivo* models (Pacheco-Alvarez et al., 2006; Yang et al., 2013; Cheng et al., 2015; Penton et al., 2016) and enables regulation of blood pressure and K^+^ homeostasis. This is most clear in the context of Familial Hyperkalemic Hypertension (FHHt), a monogenic disease in which the pathological increase in this transporter’s phosphorylation causes hypertension and hyperkalemia that are fully treatable with thiazides, the diuretics that inhibit NCC (Wilson et al., 2001).

Mutations in FHHt cause the gain-of-function of “With-No-lysine (K)” kinases 1 and 4 (WNK1 and WNK4) (Wilson et al., 2001), a pair of kinases that phosphorylate and activate the Ste20-related proline/alanine-rich kinase (SPAK) and oxidative stress responsive kinase 1 (OSR1) (Vitari et al., 2005), the enzymes that directly phosphorylate and activate NCC (Piechotta et al., 2002). WNK kinases are regulated by many mechanisms, including direct inhibitory Cl^−^ binding (Piala et al., 2014; Bazúa-Valenti et al., 2015) and ubiquitylation by the KLHL3-CUL3 E3 ligase complex (Ohta et al., 2013; Shibata et al., 2013; Wakabayashi et al., 2013; Wu and Peng, 2013; McCormick et al., 2014). They are also modulated by other kinases, such as PKA, PKC (Castañeda-Bueno et al., 2017), SGK1 (Ring et al., 2007; Rozansky et al., 2009), and tyrosine kinases (Lin et al., 2015).

The phosphorylation of NCC is not only changed in FHHt, but also in response to homeostatic challenges. For instance, hormones such as adrenergic agonists (Mu et al., 2011; Terker et al., 2014; Poulsen et al., 2021), antidiuretic hormone (ADH) (Pedersen et al., 2010; Saritas et al., 2013), and parathyroid hormone (PTH) (Penton et al., 2019), as well as changes in the extracellular concentration of electrolytes such as K^+^ (Vallon et al., 2009; Sorensen et al., 2013; Terker et al., 2015, 2016) and Ca^2+^ (Bazúa-Valenti et al., 2018; Bahena-Lopez et al., 2022a) can modify the activity of NCC through changes in its phosphorylation. Much effort has been devoted to the understanding of the kinases involved in this signaling pathway, yet comparatively little is understood of the opposite processes mediated by protein phosphatases (PPs). These enzymes catalyze dephosphorylation and, thus, inactivation of the pathway. While their importance is very clear, something as fundamental as the *bona fide* substrates of the PPs involved remain uncertain, partly due to these enzymes’ catalytic subunits apparent promiscuity, and partly due to the conflicting results provided by different *in vitro* models and the use of pharmacological unspecific inhibitors of PPs in many studies.

Potential involvement in this signaling pathway has been suggested for Protein Phosphatase 1 (PP1), Protein Phosphatase 2A (PP2A), Calcineurin (CN, also known as Protein Phosphatase 3 or 2B, PP3 or PP2B), and Protein Phosphatase 4 (PP4). PPs are presumably central in the inhibition of NCC in response to high K^+^ intake, as their inhibition clearly blunts this phenomenon (Shoda et al., 2017; Shoda et al., 2020; Kortenoeven et al., 2021). PPs are also important players in cAMP signaling transduction (Penton et al., 2019), and possibly in other signaling pathways. This review aims to summarize the evidence available for the physiological roles played by PPs in the regulation of NCC and their modulation by different stimuli, along with controversies on the physiological relevance of said mechanisms.

### Regulation by PP1

PP1 is a ubiquitous enzyme in eukaryotic cells that has been estimated to catalyze up to one-third of serine/threonine dephosphorylation reactions (Cohen, 2002). Four human genes encode PP1 isoforms: *PPP1CA* (PP1α), *PPP1CB* (PP1β), *PPP1CC* (PP1γ1), and *PPP1CCB* (PP1γ2) (Zhang et al., 1993; Cohen, 2002). These enzymes associate with other proteins to produce PP1 holoenzymes. These regulatory subunits, of which there are dozens, confer catalytic regulation, specificity, and compartmentalization of the dephosphorylation processes mediated by PP1 (Ceulemans et al., 2002; Virshup and Shenolikar, 2009). The involvement of PP1 in the regulation of NCC and the WNK-SPAK/OSR1 signaling pathway has been studied by multiple groups.

NKCC1 and NKCC2, which are the most closely related transporters to NCC in our proteome, possess a conserved PP1-binding site (Darman et al., 2001; Gagnon and Delpire, 2010). This site has been validated in NKCC1. Mutations in this site can prevent inactivation of the transporter by precluding the binding of PP1 (Darman et al., 2001; Gagnon and Delpire, 2010). However, a putative PP1 binding site is absent in NCC, which begs the question: is PP1 involved in the direct dephosphorylation of NCC?

**FIGURE 1:**
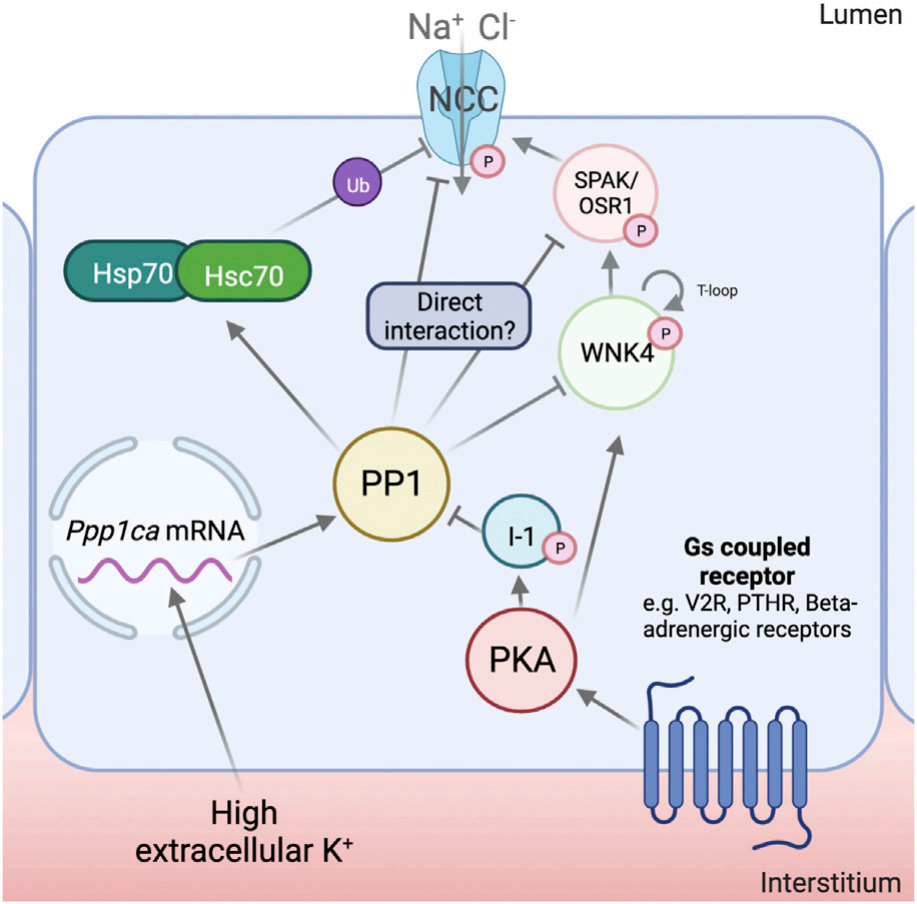
Mechanisms for NCC regulation by Protein Phosphatase 1. Protein phosphatase 1 (PP1) has been shown to increase its mRNA and protein abundance in response to high extracellular K^+^. Inhibitor-1 (I1), in response to cAMP increasing stimuli, is phosphorylated by Protein Kinase A (PKA). This, in turn, results in the inhibition of PP1 activity. PP1 has been shown to regulate With-No-Lysine (K) kinase (WNK4), Ste20-related Proline/Alanine rich Kinase (SPAK) and Na^+^-Cl^-^ Cotransporter (NCC) phosphorylation, though it is at the moment uncertain if these interactions are physiological and the degree to which they contribute to NCC regulation. PP1 has also been shown to dephosphorylate and activate the 70 kDa Heat-Shock Protein (Hsp70) and Heat-Shock Cognate 70 (Hsc70), which have been proposed to play an important role in the ubiquitylation and degradation of NCC. Created with Biorender.com.

Evidence supporting that PP1 can directly dephosphorylate NCC includes PP1-NCC binding by yeast-two-hybrid assays, coimmunoprecipitation, and phosphatase assays of PP1 on the N-terminus of NCC (Picard et al., 2014). However, experiments from our group, performed in HEK293 cells, which allow for the dissection of individual components of this signaling system (albeit, through overexpression), have recently questioned such direct effect. In our hands, PP1 regulation of NCC cannot occur directly on SPAK or NCC in these cells, but strictly requires of WNK4 to occur (Carbajal-Contreras et al., 2022) (Figure 1). Thus, direct regulation of PP1 by NCC is still in question, and could either occur only through WNK4, or depend on a regulatory subunit that mediates interaction with NCC *in vivo*. Such seems to be the case for other reported substrates of SPAK, such as CFTR and NBCe1-b, which utilize the IP_3_ receptor binding protein released with IP_3_ (IRBIT) to allow PP1 binding (Yang et al., 2011; Vachel et al., 2018).

The par excellence homeostatic challenge associated with the dephosphorylation of NCC is an increase in extracellular K^+^ (Sorensen et al., 2013). This condition increases the transcript levels of *Ppp1ca*, but not of *Ppp1cb* or *Ppp1cc*, in mouse tubule suspensions (Kortenoeven et al., 2021). Protein abundance of PP1α also increases under these conditions, as well as in the kidney cortex of mice fed a high K^+^ diet for 4 days. Oppositely, low extracellular K^+^ decreases the abundance of this enzyme. This points towards a physiological role for PP1 upon changes in extracellular K^+^ in the renal tubules. It remains to be determined whether the reported changes occur in the DCT and play a role in NCC regulation by K^+^.

An unequivocal role for PP1 in the regulation of NCC was uncovered by mice lacking I1 (Picard et al., 2014), which have reduced levels of phosphorylated NCC and blood pressure. I1, encoded by *PPP1R1A,* is a regulatory subunit of PP1 that, when phosphorylated by PKA at T35, functions as a potent, specific inhibitor of PP1 (Nimmo and Cohen, 1978). The absence of I1 was then shown to reduce the response to cAMP increasing stimuli in the DCT. The main event regulating the increase in NCC phosphorylation under these conditions was proposed to be the direct downregulation of the transporter’s dephosphorylation rate, since the increase in pNCC with forskolin/IBMX was attenuated in I1^−/−^ mice, and no changes in the phosphorylation of SPAK/OSR1 could be observed (Penton et al., 2019). However, this contrasts with findings by other groups using other cAMP-increasing stimuli, such as desmopressin (Saritas et al., 2013), where changes in SPAK/OSR1 phosphorylation could be observed. An attenuated response to beta-adrenergic agonists (which increase intracellular cAMP) has been reported in OSR1^−/−^, but not SPAK^−/−^ mice, which further supports a role for kinase activation in this pathway (Terker et al., 2014). It is also important to note that I1 is a validated substrate of CN, which could serve as a link between PP1 and this other phosphatase (Mulkey et al., 1994). CN activation could also activate PP1 and, thus, inactivate NCC indirectly.

PP1 could also modulate the activity of NCC through the dephosphorylation of Hsp70 and Hsc70, which, in this state, could induce the ubiquitylation and degradation of NCC (Kortenoeven et al., 2021). This has been shown to occur under high extracellular K^+^ conditions in tubule suspensions, and to be prevented by the specific PP1 inhibitor, tautomycetin, and by the Hsp70 inhibitor, Ver155008, but not by the PP2A and CN inhibitor deltamethrin, the CN inhibitor FK506, or the PP4 inhibitor fostriecin (Kortenoeven et al., 2021). However, Hsp70 has many molecular targets, and its inhibition could have many unintended side-effects. Also, previous work has shown that NCC phosphorylation, which is known to be diminished by high extracellular K^+^, is inversely correlated with its stability and ubiquitylation (Yang et al., 2013; Rosenbaek et al., 2014).

PP1 may also play a role in the regulation of SPAK/OSR1. Results obtained in *X. laevis* oocytes have shown PP1-dependent inhibition of SPAK that depends on other scaffolding proteins, such as AATYK or NKCC1 itself (Gagnon et al., 2007; Gagnon and Delpire, 2010). SPAK has also been shown to coimmunoprecipitate with IRBIT and PP1 in other studies, though no regulation of dephosphorylation activity towards SPAK was analyzed (Yang et al., 2011; Vachel et al., 2018).

Regarding the upstream WNK kinases, there is also evidence for interaction with PP1. WNK1’s T-loop was shown to, *in vitro*, be dephosphorylated by PP1γ, which diminishes its catalytic activity (Zagórska et al., 2007). Another work, dedicated to screening and validating novel substrates of PP1α, found a positive hit with WNK1 (Hendrickx et al., 2009). In the DCT, this interaction might only be relevant for the kidney-specific WNK1 (KS-WNK1), as the catalytically active long isoform, L-WNK1, is thought to be physiologically irrelevant in these cells (Castañeda-Bueno et al., 2012; Thomson et al., 2020; Bahena-Lopez et al., 2022b).

Finally, WNK4, the master kinase regulating NCC, has a PP1-binding site in its C-terminus that has been validated *in vitro* (Murillo-de-Ozores et al., 2018). When this site is deleted or mutated, there is a noticeable gain of function of this kinase, accompanied by an increase in the phosphorylation of its T-loop. PP1 overexpression induces the dephosphorylation of wildtype WNK4, but not of kinase mutants lacking this binding site (Murillo-de-Ozores et al., 2018). Additionally, results from our group show that forskolin-induced I1 phosphorylation induces a dramatic increase in the phosphorylation of WNK4, which is blunted in the PP1 binding-deficient WNK4 mutant. Our results in HEK293 cells show that WNK4’s dephosphorylation, but not that of NCC or SPAK, can be regulated by I1 inhibition of PP1 (Carbajal-Contreras et al., 2022). This hints towards a central role of WNK4 as a mediator of stimuli that act through this phosphatase.

Despite the previously mentioned evidence, it must be noted that PP1-mediated regulation of the WNK4-SPAK/OSR1-NCC pathway remains polemical, as negative results have also been reported. For instance, mice fed a high K^+^ gavage showed no changes in the abundance of PP1, phosphorylated (inhibited) PP1, or I1 in their kidneys; and the administration of tautomycetin (PP1 inhibitor) to these mice did not prevent the dephosphorylation of NCC (Shoda et al., 2017). This suggested that, at least in an acute setting, PP1 plays a minor role in the regulation of pNCC by K^+^. Furthermore, at least two groups found that, in kidney slices, treatment with calyculin A can indeed increase the phosphorylation of NCC, but cannot prevent the transporter’s dephosphorylation under high K^+^ conditions (Penton et al., 2016; Mukherjee et al., 2021). As will be discussed later, an effect of PP1 inhibition in kidney slices during high-K^+^ conditions was only observed in mice genetically lacking CN activity in the DCT (Banki et al., 2021). Thus, PP1 may be involved in the dephosphorylation of the WNK4 SPAK/OSR1-NCC pathway under some, but not all situations.

### Regulation by calcineurin

CN inhibitors (CNIs) are widely used in the clinic as immunosuppressants. In some patients, the side effects of CNIs resemble the alterations observed in FHHt, including hypertension, hyperkalemia, metabolic acidosis and hypercalciuria. Thus, several groups have tested the effects of CNIs on the activity of NCC in rodents. Treatment with CNIs, including tacrolimus (or FK506) and cyclosporin, produces FHHt-like alterations and promotes NCC phosphorylation (Hoorn et al., 2011; Melnikov et al., 2011). Interestingly, the hypertensive effect of tacrolimus treatment was not observed in NCC knockout mice or in thiazide-treated mice, suggesting a central role for NCC modulation in the development of these side effects. The effects of tacrolimus in NCC phosphorylation, plasma K^+^, and blood pressure levels were attenuated in tubule specific FKBP12^−/−^ mice (Lazelle et al., 2016). FKBP12 is the 12 kDa FK506-binding protein that inhibits CN when bound to tacrolimus. Thus, it was shown that the effects of tacrolimus on NCC are mediated by direct inhibition of CN in the renal epithelium.

These observations have been extended to patients treated with CNIs. Increased urinary vesicle abundance of NCC and pNCC in patients treated with CNIs has been observed (Tutakhel et al., 2017). This has also been shown to correlate with increased blood pressure (Rojas-Vega et al., 2015) and with thiazide responsiveness (Tutakhel et al., 2017). While a greater response to thiazides has been observed for patients treated with tacrolimus (Hoorn et al., 2011), an actual benefit for NCC blockade with thiazides compared to other antihypertensive drugs remains controversial (Moes et al., 2017), and further studies are required to test this hypothesis in the clinical setting.

**FIGURE 2:**
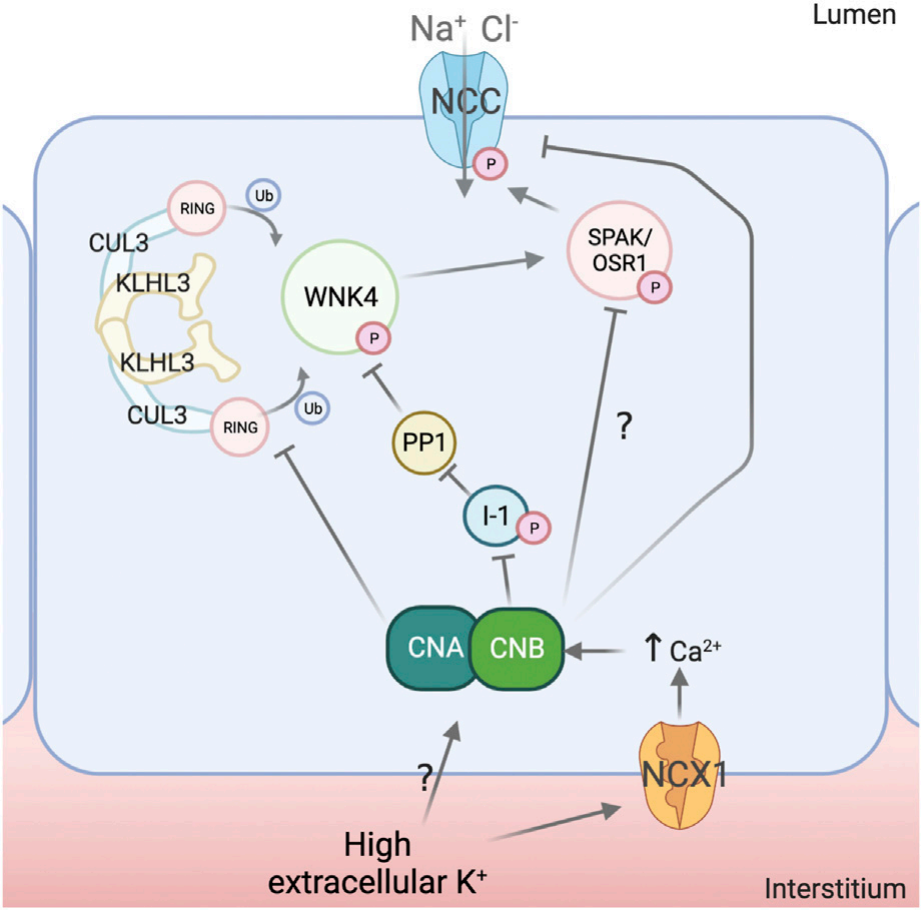
Mechanisms for NCC regulation by calcineurin. Calcineurin (CN) activity has been implicated in the dephosphorylation of the Na^+^-Cl^-^ Cotransporter (NCC) in response to high extracellular K^+^. Modulation of the Na^+^-Ca^2+^ exchanger (NCX1) activity by K^+^ has been postulated as a mechanism to induce changes intracellular Ca^2+^ concentration and thus promote CN activation, although this mechanism requires confirmation and additional mechanisms may also participate. Evidence suggests that CN can directly dephosphorylate NCC and also affect upstream elements of the signaling pathway. For instance, CN appears to dephosphorylate Kelch-Like 3 (KLHL3), which leads to increased ubiquitylation of With-No-Lysine (K) kinase 4 (WNK4) and Kidney-Specific With-No-Lysine (K) kinase 1 (KS-WNK1) and, thus, decreased activation of Ste20-related Proline/Alanine rich Kinase (SPAK) and Oxidative Stress Responsive kinase 1 (OSR1). Inhibitor 1 (I1) has also been shown to be a substrate of CN and, consequently, this protein may function as a node linking CN activation to Protein phosphatase 1 (PP1) activation, with the implications that this may have in the DCT (see Figure 1). Created with Biorender.com.

The effects of CNIs on NCC in animal models are accompanied by changes in the expression or phosphorylation levels of known regulators of NCC activity. For instance, increased renal expression levels of WNK4 (Hoorn et al., 2011; Melnikov et al., 2011; Ishizawa et al., 2019), WNK1 (Ishizawa et al., 2019), SPAK (Hoorn et al., 2011), and increased phosphorylation of SPAK (Ishizawa et al., 2019) and KLHL3 (Ishizawa et al., 2019) have been observed. Changes in abundance of WNK kinases may be secondary to changes in phosphorylation of KLHL3 at S433, given that phosphorylation of this site has been shown to prevent binding to WNK kinases (Shibata et al., 2014). This phosphorylation was shown to be modulated by CN in *in vitro* experiments (Ishizawa et al., 2019). Moreover, KLHL3-S433 phosphorylation was shown to increase in mice on a low K^+^ diet (Ishizawa et al., 2016) and to decrease in response to high extracellular K^+^ in an *in vitro* cell model (Ishizawa et al., 2019). This latter effect was prevented by tacrolimus. Accordingly, it was proposed that high extracellular K^+^ may activate CN to promote KLHL3 dephosphorylation, leading to decreased WNK4 and WNK1 levels (Figure 2).

There is also evidence of direct dephosphorylation of NCC by CN. Lazelle et al. showed that in cells expressing constitutively active SPAK, dephosphorylation of NCC induced by depolarization with BaCl_2_ was prevented with tacrolimus (Lazelle et al., 2016). Shoda et al. proposed that CN mediates direct dephosphorylation of NCC in response to acute increases in extracellular K^+^ (Shoda et al., 2017). In mice that were given an oral K^+^ load *via* gavage, rapid NCC dephosphorylation was completely prevented by pre-treatment with tacrolimus or with the calmodulin inhibitor W7. No changes in WNK4, SPAK or pSPAK were observed, suggesting a direct effect on NCC phosphorylation. However, this conclusion must be taken with reserve given that, in another study with a similar experimental setting, no measurable decrease in pSPAK/OSR1 levels was observed through Western blot of whole kidney lysates, but could be clearly noted in DCT cells by immunofluorescence (Mukherjee et al., 2021).

Given that CN is regulated by Ca^2+^, a link between high extracellular K^+^ and intracellular Ca^2+^ was proposed to be mediated by the Na^+^-Ca^2+^ exchanger NCX1, expressed in the basolateral membrane of the DCT. This antiporter might work in reverse mode and transport Ca^2+^ into the cell with high K^+^-induced depolarization of the DCT (Shoda et al., 2020). In HEK293 cells, Ca^2+^ and CN-dependent dephosphorylation of NCC in response to high extracellular K^+^ was observed. This was prevented by knockdown of NCX1 or by treatment with the NCX1 inhibitor SEA0400. Similarly, in mice, administration of SEA0400 1 hour prior to an acute oral K^+^ load prevented NCC dephosphorylation. In contrast to the results obtained in the aforementioned *in vivo* experiments, tacrolimus was unable to prevent NCC dephosphorylation in kidney slices incubated in high K^+^ solutions. It remains to be shown, however, if tacrolimus can effectively inhibit CN in this system, as low drug permeability may be a plausible explanation for these inconsistencies. Supporting this, it was shown that treatment with tacrolimus of kidney tubules in suspension results in a clear increase in NCC phosphorylation (Tutakhel et al., 2017). Moreover, genetic disruption of CN activity coupled to pharmacologic disruption of PP1 activity significantly prevented NCC dephosphorylation by high K^+^ in kidney slices (Banki et al., 2021).

Finally, the role of CN in NCC regulation was more recently addressed with an inducible, DCT-specific knockout mouse model for the B1 subunit of CN (Banki et al., 2021). CN normally functions as a heterodimer formed by an A subunit that provides catalytic activity and a calmodulin-binding site, and a Ca^2+^-binding regulatory B subunit. Both subunits are necessary for full phosphatase activity. Whereas the A subunit is encoded by three separate genes (*PPP3CA, PPP3CB*, and *PPP3CC*), the B subunit is encoded by two genes (*PPP3R1* and *PPP3R2*). Only the B1 isoform (CaNB1, encoded by *PPP3R1*) appears to be normally expressed in the DCT (Chen et al., 2021). Thus, by knocking out CaNB1 in the DCT, CN activity was disrupted.

Surprisingly, 3 weeks after disruption of CaNB1 only hypomagnesemia and metabolic acidosis were observed, but no hypertension, hyperkalemia, hypercalciuria, or increased pNCC levels. Hypomagnesemia was associated with decreased expression of proteins involved in transcellular Mg^2+^ transport. Several DCT-enriched molecules were downregulated at the mRNA and protein levels, like NCC, TRPM6, KS-WNK1, and specially parvalbumin, whose mRNA and protein levels were drastically reduced. Interestingly, pNCC levels were similar to those of wild type animals, despite lower total NCC levels, suggesting that reduction of CN activity may indeed lead to increased NCC phosphorylation. Supporting this, tacrolimus treatment did not increase pNCC levels in the knockouts. It was proposed that the discrepancy between the effects observed with genetic disruption of CaNB1 and pharmacologic inhibition of CN could be due to either incomplete suppression of CN activity by drugs or to the length of the suppression of activity, as most previous pharmacologic studies were performed with treatment in shorter timeframes (Hoorn et al., 2011; Lazelle et al., 2016; Shoda et al., 2017; Ishizawa et al., 2019). It was concluded that disruption of CN activity strongly affects the transcriptome and the proteome of the DCT.

### Regulation by PP2A and PP4

PP2A was proposed to regulate the activity of NKCC1 through work done in Calu-3 cells (Liedtke et al., 2005). This has not been explored in further works.

PP4 overexpression in *X. laevis* oocytes reduced NCC activity. The use of NCC phosphoablative or phosphomimetic mutants abolished this effect (Glover et al., 2010). It was not explored if this occurs directly or through upstream mediators. This work also showed enriched PP4 expression in the DCT through immunohistochemistry, although this is not evident at the transcript level (Chen et al., 2021). The physiological relevance of these findings has not been further tested.

## Concluding remarks

The works reviewed here address the role of serine-threonine phosphatases in the signaling pathways implicated in NCC modulation and particularly highlight PP1 and CN as relevant modulators in these pathways. However, more physiologically accurate models ought to be studied to better understand the actual relevance of some of the findings. For instance, *knock-in* models for protein phosphatases’ binding sites of proposed interactors, or unbiased techniques with samples derived from DCT cells to study the true interactors of PPs involved in NCC regulation could allow further dissection of the pathways. In addition, it is very likely that the PPs reviewed here play other yet unknown roles in the cell biology of the DCT. These other roles are also likely to influence epithelial transport processes and thus, their characterization will be necessary to fully understand the impact of PPs activity in the function of the DCT.
